# Supplementary material for: Vitamin D Modified DSS-Induced Colitis in Mice via STING Signaling Pathway
Source: Biology (Basel). 2025 Jun 18;14(6):715. doi: 10.3390/biology14060715 (PMC12190092; doi:10.3390/biology14060715)
Supplement: Supplementary file 1 [file biology-14-00715-s001.zip › Supplementary Materials S1: 16s-report of gut microbiota/Groups/diff_analysis/metagenomeRSpecies/figure/barplot/top20log.pdf]

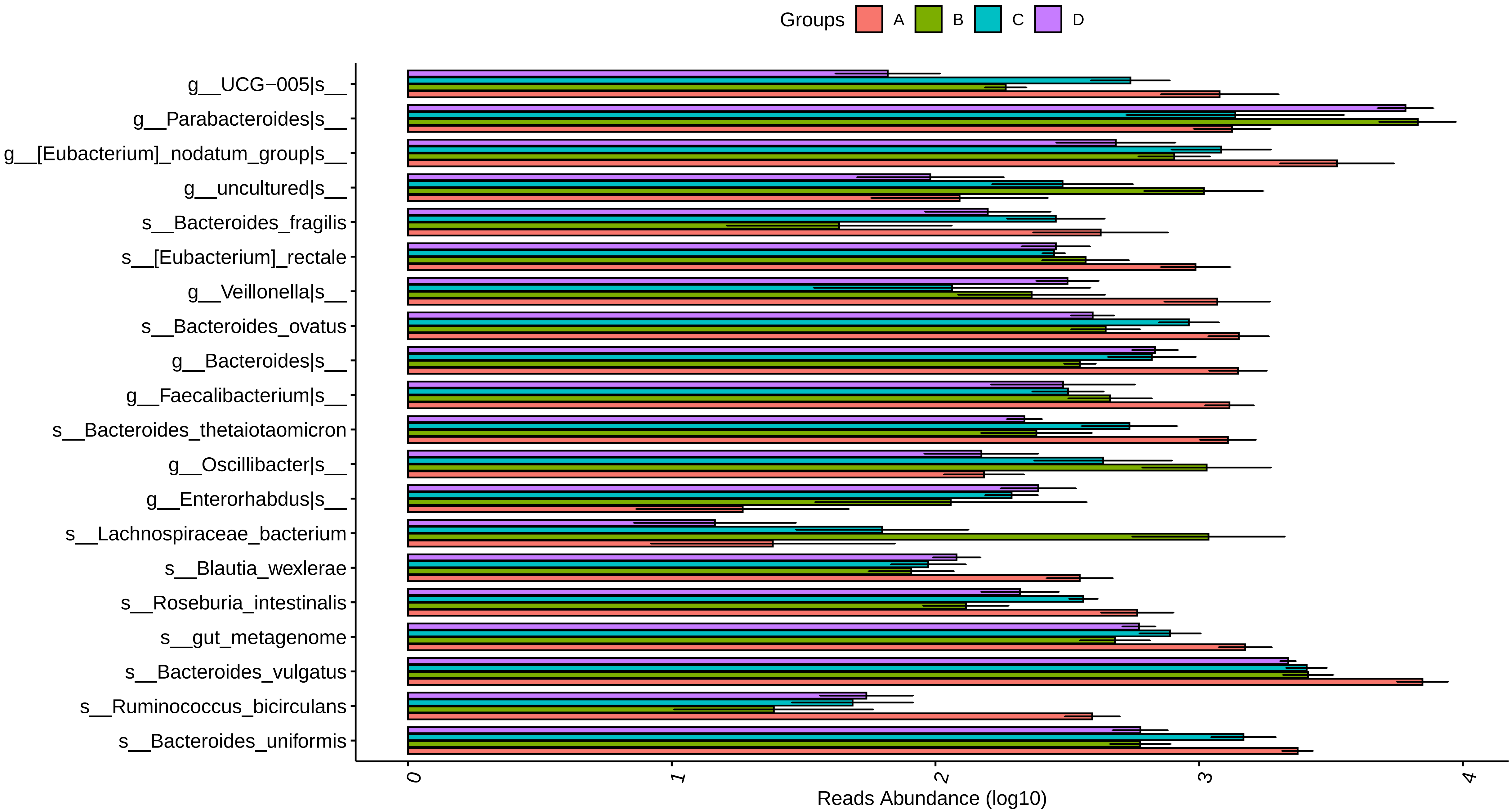

Groups
A
B
C
D
g__UCG−005|s__
g__Parabacteroides|s__
g__[Eubacterium]_nodatum_group|s__
g__uncultured|s__
s__Bacteroides_fragilis
s__[Eubacterium]_rectale
g__Veillonella|s__
s__Bacteroides_ovatus
g__Bacteroides|s__
g__Faecalibacterium|s__
s__Bacteroides_thetaiotaomicron
g__Oscillibacter|s__
g__Enterorhabdus|s__
s__Lachnospiraceae_bacterium
s__Blautia_wexlerae
s__Roseburia_intestinalis
s__gut_metagenome
s__Bacteroides_vulgatus
s__Ruminococcus_bicirculans
s__Bacteroides_uniformis
0
1
2
3
4
Reads Abundance (log10)
